# *CD46* Gene Editing Confers Ex Vivo BVDV Resistance in Fibroblasts from Cloned Angus Calves

**DOI:** 10.3390/v17060775

**Published:** 2025-05-29

**Authors:** Aspen M. Workman, Michael P. Heaton, Brian L. Vander Ley

**Affiliations:** 1 US Meat Animal Research Center, USDA, Agricultural Research Service, Clay Center, NE 68933, USA; mike.heaton@usda.gov; 2 Great Plains Veterinary Educational Center, University of Nebraska–Lincoln, Clay Center, NE 68933, USA; bvanderley2@unl.edu

**Keywords:** angus, bovine viral diarrhea virus, BVDV, cattle, CD46, cloning, CRISPR, disease resistance, gene editing

## Abstract

A previous study demonstrated that a 19-nucleotide edit, encoding a six amino acid substitution in the bovine *CD46* gene, dramatically reduced bovine viral diarrhea virus (BVDV) susceptibility in a cloned Gir (*Bos indicus*) heifer. The present study aimed to replicate this result in American Angus (*Bos taurus*) using genetically matched controls and larger sample sizes. CRISPR/Cas9-mediated homology-directed repair introduced the identical *CD46* edit, encoding the A_82_LPTFS amino acid sequence, into exon 2 of *CD46* in primary Angus fibroblasts. Thirty-three cloned embryos (22 *CD46*-edited and 11 unedited) were transferred to recipient cows. However, all pregnancies resulted in pre- and perinatal losses due to cloning-related abnormalities, preventing in vivo BVDV challenge. Consequently, ex vivo BVDV susceptibility assays were performed on primary fibroblast cell lines rescued from deceased cloned Angus calves. Infection studies revealed significantly reduced susceptibility in the edited lines, comparable to the resistance previously observed from the edited Gir heifer. These studies extend the applicability of this finding from Gir to the most common US beef breed, Angus, suggesting the potential for broad application of *CD46* editing in BVDV control. Continued advancements in cloning technology will enhance the potential of gene-editing for producing disease-resistant livestock.

## 1. Introduction

Gene-editing technologies are rapidly advancing and provide novel tools for producing disease-resistant livestock [1]. Unlike traditional selective breeding, which restricts trait improvement to existing genetic variation, gene editing allows for the introduction of new traits by creating novel genetic variants. This is especially valuable for enhancing disease resistance when natural resistance alleles are not available. This strategy was recently demonstrated in a 2023 proof-of-concept study that produced the first gene-edited bovine with genetic resistance to bovine viral diarrhea virus (BVDV), a major pathogen responsible for respiratory, gastrointestinal, and reproductive diseases that cost the cattle industry approximately $2 billion annually [2,3].

The genetic modification strategy for BVDV focused on disrupting the viral entry mechanism by targeting the primary viral receptor, CD46 [4]. CRISPR/Cas9-mediated homology-directed repair was used to replace 19 nucleotides within exon 2 of the *CD46* gene in primary bovine fibroblasts from a Gir breed (indicine) heifer [2]. The biallelic modification resulted in the expression of a novel CD46 receptor variant in the edited cells. This variant contained a six amino acid substitution (G_82_QVLAL to A_82_LPTFS) within the extracellular protein domain responsible for BVDV binding, thereby preventing viral entry [5]. The edited cells were used for somatic cell nuclear transfer (SCNT) to produce embryos, which were transferred into recipient cows.

A single *CD46*-edited Gir heifer calf (“Ginger”) was born live and healthy in 2021 and was subsequently challenged with BVDV at 10 months of age. Ginger displayed a significant reduction in viral susceptibility as measured by reduced clinical signs and a lack of measurable infectious virus in her blood [2]. Significantly, Ginger has remained healthy for the first three years of her life, displaying no obvious adverse physical effects. Additionally, her reproductive capabilities have not been compromised by the edit when fertilization occurs with semen from an unedited bull [6]. Ginger’s subsequent pregnancy and delivery of a live, healthy calf in December 2024 further demonstrated that the *CD46* edit does not negatively impact the ability to carry a pregnancy to term. This research demonstrates the potential of gene editing to address longstanding challenges in livestock disease management, opening new avenues for improving animal health and reducing economic losses in the cattle industry.

Here, our initial goal was to produce male and female American Angus calves to study the BVDV resistance conferred by the *CD46* A_82_LPTFS edit in a popular Taurine beef breed. However, the female primary fibroblast cell line showed signs of significant deterioration prior to SCNT and was thus excluded. Despite the apparently normal appearance of the male primary fibroblast cell line, significant cloning-related abnormalities prevented the birth of live, healthy calves for in vivo BVDV challenge. Therefore, primary skin fibroblast cell lines were established from four cloned calves (two each *CD46*-edited and unedited) for ex vivo infection studies. The results presented here demonstrate that the *CD46* gene edit, previously shown to significantly reduce BVDV susceptibility in a cloned Gir heifer, confers similar levels of resistance in primary skin fibroblasts from Angus, the most common U.S. beef breed.

## 2. Materials and Methods

### 2.1. Ethics Statement

All protocols for reproductive cloning, fetal tissue collection, and birthing were reviewed and approved by the Institutional Animal Care and Use Committee (IACUC) of Trans Ova Genetics (TOG, Sioux Center, IA, USA; (Project ID 182)). Protocols for blood collection and tissue biopsies from purebred American Angus were reviewed and approved by the US Meat Animal Research Center (USMARC) IACUC (Project ID 194).

### 2.2. Choice of Breed and Selection of Animals

We selected the American Angus beef breed due to its popularity (>330,000 registered animals, >60% of processed beef) and availability for study at the USMARC (Clay Center, NE, USA) [7]. Low-coverage whole genome sequence (WGS) data (~0.5–1× coverage) were available from a USMARC resource population developed for studying natural, loss-of-function alleles [7]. Using imputed genotypes derived from this WGS data, we identified unrelated male and female American Angus calves with the desired low-risk genotypes for bovine congestive heart failure (BCHF): the ‘CC’ genotype at both the *ARRDC3* locus (BovineHD0700027239; rs109901274; BCHF5) and the *NFIA* locus (BovineHD0300024308; rs133192205: BCHF32) [8]. From these, a weaned bull calf (“Banner”, USMARC no. 20218042) and a heifer calf (“Olivia”, USMARC no. 20218209) were chosen from different sire lines to minimize their pedigree relationship.

### 2.3. WGS, Alignment, and SNP Genotyping

Banner and Olivia’s genotypes were confirmed with 15× WGS from whole blood samples. Unless otherwise indicated, reagents were molecular-biology grade. DNA from blood or cell lines was extracted with standard procedures that used RNase and protease digestion, phenol/chloroform extraction, and ethanol precipitation [9]. Purified DNAs were dissolved in a solution of 10 mM TrisCl, 1 mM EDTA (TE, pH 8.0), and stored at 4 °C. For WGS, 2 μg of genomic DNA was fragmented and used to make indexed, 500 bp, paired-end libraries [10]. Dynamic pools of indexed libraries were sequenced with massively parallel sequencing machines (either NextSeq500 or NextSeq2000, Illumina Inc., San Diego, CA, USA) with the appropriate kits producing 2 × 150 bp paired-end reads. Pooled samples were sequenced until a minimum of 40 Gb of data was collected with greater than Q20 quality for each animal, thereby producing at least 13.3-fold mapped read coverage for each index (90% of reads mapped to a 2.7 Gb reference genome). This level of coverage provides scoring rates and SNP genotype accuracies that exceed 99% [11]. Raw FASTQ files are available in the NCBI SRA under accession numbers SRR33165226—SRR33165235, BioProject accession number PRJNA1245396 (BioSamples SAMN47753601, SAMN47754587, SAMN47755051, and SAMN48016586-SAMN48016592) in the NCBI BioProject database. Using a Linux command line environment, genotypes for the sequences of interest were identified and counted in compressed FASTQ files by decompressing on-the-fly with gunzip -c and searching the resulting data stream for case-insensitive sequences (grep -ie). The total number of lines containing sequences was concurrently counted using wc -l. An example of the entire command is as follows: gunzip -c [*library filename*]*|grep --color = always -ie [*seq1*] -e [*seq2*]|tee >(wc -l). The search sequences used were: wild type GQVLAL (5′-CCAGTAACTCCTGGTCAAGTCCTGGCTCTCGTTTGTCAG-3′ and 5′-CTGACAAACGAGAGCCAGGACTTGACCAGGAGTTACTGG-3′); edited ALPTFS-“c” (5′-CCAGTAACTCCGGCATTGCCTACcTTCAGTGTTTGTCAG-3′ and 5′-CTGACAAACACTGAAgGTAGGCAATGCCGGAGTTACTGG-3′); and ALPTFS-“a” (5′-CCAGTAACTCCGGTTTGTCAG-3′ and 5′-CTGACAAACACTGAAtGTAGGCAATGCCGGAGTTACTGG-3′).

To align WGS data to a bovine reference genome assembly, FASTQ files were aggregated by animal for removal of adapter sequences and low-quality bases by Cutadapt version 4.0, FASTQC, and wrapper tool TrimGalore version 0.6.10. Sets of trimmed reads for each animal were aligned to the Hereford bovine reference genome (ARS-UCD1.2) with the Burrows–Wheeler Alignment tool (BWA) aln algorithm version 0.7.12 [12]. The files were merged and collated with the BWA sampe command, and the resulting sequence alignment map (SAM) files were converted to binary alignment map (BAM) files and sorted with SAMtools version 1.3.1 [13]. PCR duplicates were marked in the BAM files with the Genome Analysis Toolkit (GATK) version 3.6 [14]. The GATK module RealignerTargetCreator was used to identify regions with small indels, and those regions were realigned with the IndelRealigner module. The BAM files produced at each step were indexed with SAMtools and made available via object storage through a USDA, ARS, USMARC website interfacing with a cloud storage provider (Amazon Web Services, Inc. Seattle, WA, USA). The latter allows any of these animals’ genomic DNA sequence to be viewed in a user-friendly environment, such as the Integrated Genome Viewer (IGV). This public, livestock whole genome resource facilitates in silico identification of functional variants and provides a means of translating WGS data into a practical biological and evolutionary context for generating and testing hypotheses.

### 2.4. Tissue Biopsies and CRISPR/Cas9 Editing of Primary Fibroblasts

Ear punch tissue biopsies were collected from Banner and Olivia (Allflex tissue sampling unit) for primary skin fibroblast isolation at TOG. The resulting primary skin fibroblast cell lines were given IDs TO807 (Banner) and TO808 (Olivia). CRISPR/Cas9-mediated homology-directed repair (HDR) was used to introduce a 19-nucleotide in-frame substitution, encoding a six amino acid substitution, in exon 2 of the bovine *CD46* gene in the two primary fibroblast cell lines, as previously described [2] (Recombinetics Inc, Egan, MN, USA). Briefly, cells were maintained at 37 °C, 5% CO_2_ in DMEM supplemented with 20% irradiated fetal bovine serum, 100 I.U./mL penicillin and streptomycin. Actively growing cells were prepared for transfection (Neon Transfection System, Life Technologies, Carlsbad, CA, USA), as previously described. The guide RNA was identical to that previously used (5′-ACGAGAGCCAGGACTTGACC-3′). A pool of five distinct single-stranded oligonucleotide donors (ssODNs) was used to distinguish biallelic HDR. Four of these ssODNs each contained a different synonymous single nucleotide polymorphism (SNP). The five ssODNs used here were: 1) C*C*TCCTGGAGAGACGACCATGTATTATTATCCTGACAAAC-ACTGAATGTAGGCAATGCCGGAGTTACTGGCTGGAAACCCAGATGACATTCATACACA*A*T; 2) C*C*TCCTGGAGAGACGACCATGTATTATTATCCTGACAAACACTGAA**G**GTAGGCAATGCCGGAGTTACTGGCTGGAAACCCAGATGACATTCATAC-ACA*A*T; 3) C*C*TCCTGGAGAGACGACCATGTATTATTATCCTGACAAACACTGAATGTAGGCAA**AG**CCGGAGTTACTGGCTGGAAACCCAGATGACATTCATAC-ACA*A*T; 4) C*C*TCCTGGAGAGACGACCATGTATTATTATCCTGACAAACACTGAATGT**T**GGCAATGCCGGAGTTACTGGCTGGAAACCCAGATGACATTCATAC-ACA*A*T; and 5) C*C*TCCTGGAGAGACGACCATGTATTATTATCCTGACAAACACT**A**AATGTAGGCAATGCCGGAGTTACTGGCTGGAAACCCAGATGACATTCATAC-ACA*A*T. In these sequences, the asterisk denotes phosphorothioated base, the underline denotes the bases introducing the CD46 A_82_LPTFS substitution, and the bold and highlighted letters denote the synonymous SNPs.

### 2.5. Genotyping Clones Derived from Single Cells

Edited fibroblasts were screened for homozygous CD46 A_82_LPTFS substitution by restriction fragment length polymorphism (RFLP) of PCR products (AccuStart II GelTrack PCR SuperMix, Quanta Bio, Beverly, MA, USA) produced with oligonucleotides: btCD46 NJ F1 (5′-TTCTCCAACAGGCCAGAAGC-3′) and btCD46 NJ R1 (5′-AGGCAACCAATCG-TGACGAA-3′). PCR cycling conditions were as follows: initial denaturation at 95 °C for 2 min, followed by 35 cycles of 95 °C for 20 s, 62 °C for 20 s, and 72 °C for 45 s, with a final extension at 72 °C for 5 min. PCR amplicons were digested with MspI (New England Biolabs; Ipswich, MA, USA) and separated by agarose gel electrophoresis. Clones identified as homozygous by RFLP were confirmed by analyses of custom Sanger sequencing.

### 2.6. SCNT and Reproductive Cloning

**Figure 1:**
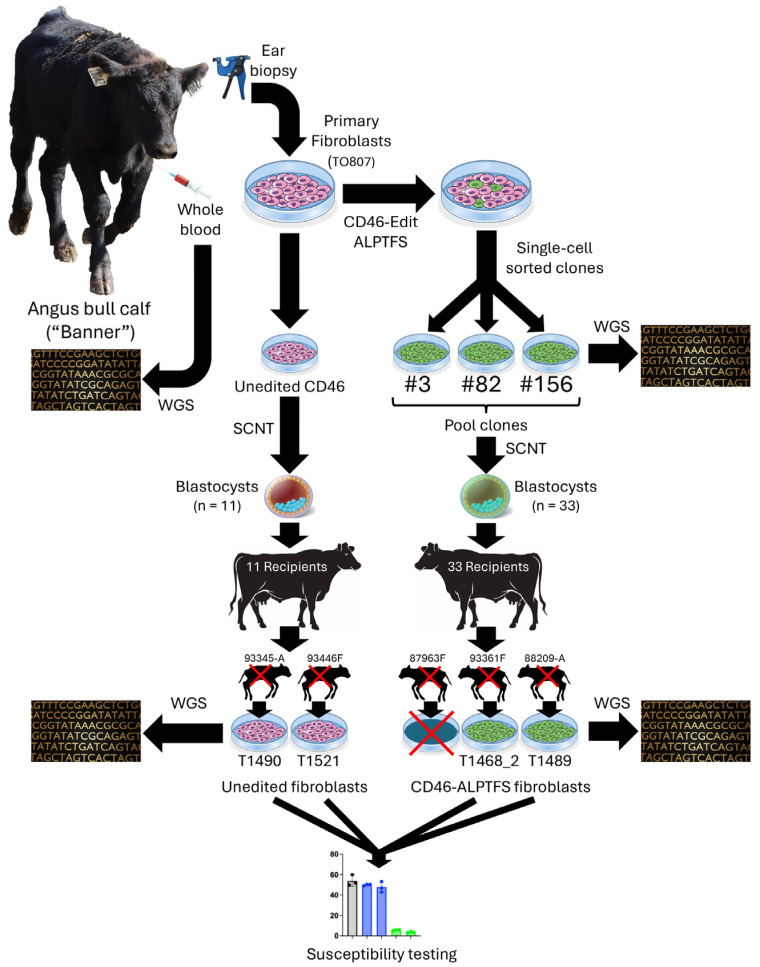
A schematic overview of the experimental flow used with Banner, from biopsy to ex vivo BVDV challenge. WGS, whole genome sequence; SCNT, somatic cell nuclear transfer.

Reproductive cloning by SCNT was contracted to TOG and performed as previously described [15,16]. Following microscopic evaluation of the *CD46*-edited skin fibroblasts, the quality of the *CD46*-edited female Angus (Olivia) fibroblast cell line was deemed insufficient for SCNT and was therefore excluded from further experimentation. Consequently, SCNT was performed exclusively with the male Angus (Banner) fibroblast clones confirmed to be homozygous for the *CD46* gene-edit encoding the A_82_LPTFS substitution, alongside wild-type, unedited Banner controls. Grade 1 Banner embryos containing either the unedited *CD46* (*n* = 11) or the *CD46* gene-edit (*n* = 22) were implanted into each of 33 recipient cows. Pregnancies were confirmed by ultrasound at 40 days post-transplantation. The average gestation length for American Angus cattle is 283 days. The experimental design, detailing the process used to create and test *CD46* gene edited bovine fibroblasts for BVDV resistance in the Banner calf, is graphically summarized in Figure 1.

### 2.7. Necropsy and Histopathological Evaluation of Cloned Calves

Aborted calves, and those that died within hours of birth, were transported to the Nebraska Veterinary Diagnostic Center (NVDC) at the University of Nebraska–Lincoln for evaluation. Comprehensive pathology reports were generated, including both gross necropsy findings and histopathological examination. Findings were compared between edited and unedited clones to better understand the causes of abortion and neonatal death in calves, with particular attention to potential cloning-related abnormalities affecting multiple organ systems.

### 2.8. Isolation of Primary Skin Fibroblasts from CD46 Edited and Unedited Controls

Primary skin fibroblasts were isolated from *CD46* edited and unedited control fetuses/calves at TOG. Primary skin fibroblasts were grown in DMEM supplemented with 15% irradiated fetal bovine serum (Atlas Biologicals, Fort Collins, CO, USA) and 1× antibiotic-antimycotic (Gibco, Grand Island, NY, USA).

### 2.9. Evaluating CD46 DNA Sequences of Fibroblast Cell Lines with 15× WGS

Aligned genomes, generated as described in the Methods section entitled “WGS, alignment, and SNP genotyping,” were viewed with the Integrative Genomics Viewer (IGV) version 2.12.2 by selecting the ARS-UCD1.2 (“bos Tau9”) reference genome and loading this session file URL: https://s3.us-west-2.amazonaws.com/usmarc.heaton.public/WGS/AngusCD46/ARS1.2/sessions/AngusBanner_CD46_10tracks.xml (accessed on 5 March 2025). The genome for the male Angus donor calf Banner and its unedited and edited fibroblast cell lines and reproductively cloned brother calves were viewed together with the Gir cell line (TO470) and the edited Gir (Ginger) in IGV. The genomic DNA sequences in a 150-kb region centered on the *CD46* gene were visually inspected in IGV for differences at the nucleotide level. The edited site encoding CD46 amino acid residues 82 to 87 (ARS-UCD1.2; ch16:75,617,415 to ch16:75,617,432) was also inspected. Potential off-target sites were searched for in the ARS-UCD1.2 bovine reference genome with Cas-OFFinder (version 2.4) [17] and the target gRNA sequence: 5′-ACGAGAGCCAGGACTTGACC-3′. Using PAM type SpCas9 from Streptococcus pyogenes (5′-NGG-3′) and the *Bos taurus* reference assembly ARS UCD1.2, off-target edits were a search for zero, one, and two mismatches and bulges, and those sites were manually inspected in IGV for the aligned genomes.

### 2.10. Infection and Flow Cytometric Detection of BVDV in Primary Skin Fibroblasts from CD46 Edited and Unedited Controls

For infection studies, primary fibroblasts were seeded in 24-well plates at a density of 1.65 × 10^5^ cells per well and incubated 24 h at 37 °C with 5% CO_2_. The following day, cells were washed four times with DMEM and infected with BVDV isolates or serum from cattle persistently infected with BVDV. Following a 2 h incubation, cells were washed four times with PBS to remove unbound virus, and cells were incubated in DMEM supplemented with 5% horse serum (ATCC, Manassas, VA, USA) at 37 °C for 20–72 h, as described in the figure legends. BVDV infection efficiency was determined by flow cytometric detection of BVDV E2 glycoprotein, as previously described [2]. To block heparan sulfate binding sites on the virus envelope proteins, virus dilutions were pre-incubated with 200 µg/mL heparin (H3149-10KU, Sigma-Aldrich, St. Louis, MO, USA) at 37 °C for 30 min. Virus infections were then conducted as described above.

### 2.11. Immunofluorescence Staining and Flow Cytometric Detection of Bovine CD46

Cellular localization of the CD46 protein was visualized by immunofluorescence staining and quantified by flow cytometry using a custom made polyclonal anti-bovine CD46 antibody, as previously described [2].

## 3. Results

### 3.1. CD46 Editing of Primary Skin Fibroblasts

**Figure 2:**
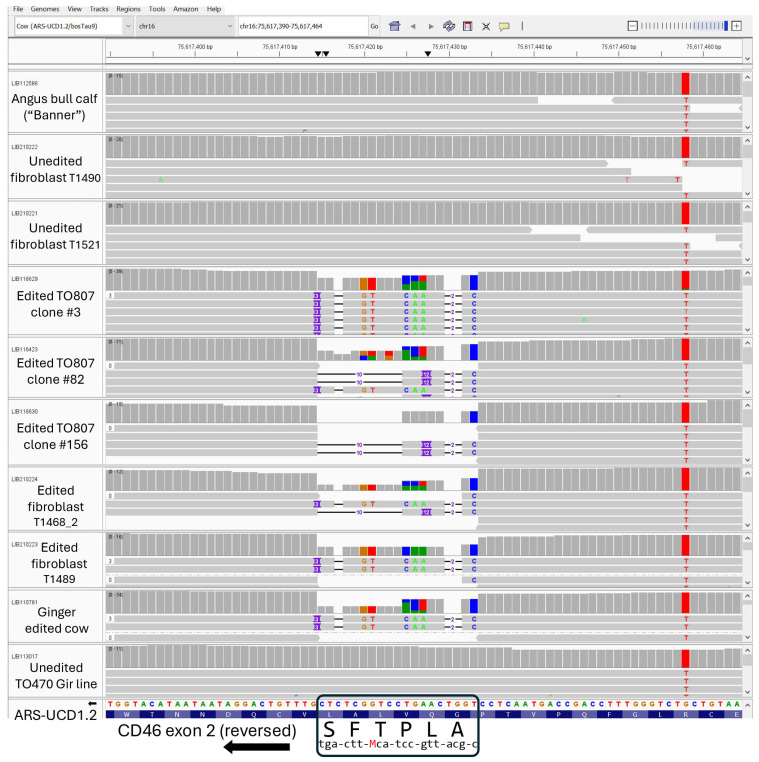
Genomic sequences viewed in IGV software showing the bovine *CD46* region of exon 2 encoding the A_82_LPTFS_87_ substitution in edited cell lines and cloned animals. The annotated screenshot shows a 76 bp region of genomic sequence aligned to bovine reference assembly ARS-UCD2.1. The original *CD46* edit had an intentional synonymous a/c (“M”) SNP in the T85 codon for screening against cell lines having one allele with the correct HDR edit, and the other allele deleted due to NHEJ activity (hemizygous genotype). Consequently, the two distinct edited 19 bp alleles can be distinguished from each other when reads are aligned to a reference sequence assembly. IGV Session URL: https://s3.us-west-2.amazonaws.com/usmarc.heaton.public/WGS/AngusCD46/ARS1.2/sessions/AngusBanner_CD46_10tracks.xml (accessed on 5 March 2025).

CRISPR/Cas9-mediated HDR was used to introduce a precise 19-nucleotide substitution in exon 2 of the *CD46* gene. This modification resulted in a six amino acid substitution (G_82_QVLAL to A_82_LPTFS) within the BVDV binding domain of CD46. A total of 480 single-cell colonies were screened for biallelic modification using RFLP analysis. In the male fibroblast line (TO807), 105 out of 480 colonies (21.9%) were homozygous for HDR. Based on homozygous-positive RFLP results, cellular morphology, and growth rate, 36 single-cell clones were selected for further sequence analysis. Sanger sequencing revealed that 25 of 36 clones (69.4%) contained the desired biallelic modification. Three single-cell clones (#3, #82, and #156) were selected for WGS to verify the structural integrity of the complete *CD46* gene locus (Figure 2). Sequence confirmed single-cell clones were then pooled for reproductive cloning.

### 3.2. Reproductive Cloning Outcomes

Thirty-three Grade 1 male embryos (22 *CD46*-edited and 11 unedited) were transferred into 33 recipient cows seven days after SCNT and in vitro culture. By day 40 post-SCNT, 15 of 22 *CD46* edited and six of 11 unedited pregnancies were established. By day 200, three fetuses from each group remained. Thereafter, two of the three *CD46* edited fetuses were aborted at 242- and 246-days gestation. The remaining *CD46* edited fetus was stillborn after induction at 275 days gestation. In the unedited control group, one pregnancy was lost prior to 275 days; however no fetal remains were observed or recovered. Of the remaining two control pregnancies, one resulted in a live birth at 277 days (induced) and the other at 283 days (natural calving); however, both control calves succumbed to cloning-related abnormalities within hours of birth (Table 1). Necropsy findings in cloned calves revealed complex syndromes affecting multiple organ systems, including skeletal, muscular, and hepatic abnormalities (Supplementary File S1). Notably, all calves exhibited weights significantly higher than the typical range for birth weights for American Angus calves (70–90 lbs), with weights ranging from 98 to 122 lbs at term (Table 1). This increased birth weight is consistent with large offspring syndrome, a well-documented complication of SCNT cloning [18]. These issues were present in both *CD46* edited and unedited cloned fetuses/calves. Thus, the use of fibroblasts for editing and cloning of two selected Angus calves did not produce healthy, live cloned calves in this effort, regardless of whether they had the *CD46* gene edit or not.

### 3.3. Search for Off-Target Edits in CD46 Edited Calves

Primary skin fibroblast cell lines were successfully established from two of three *CD46*-edited calves and both unedited control calves (Table 1). WGS analyses of the resulting cell lines confirmed the intended biallelic modification of the *CD46* gene, showing the expected 19 nucleotide *CD46* edit encoding the A_82_LPTFS sequence (visualized in Figure 2), with independent allele counts detailed in Table 2. Except for the 19 nucleotide edit, the 42-kb region spanning *CD46* was intact and identical in all samples, including the donor cell line (Banner), which itself is homozygous at every base for this entire gene region and more than one Mb upstream. The nearest heterozygous SNP in Banner was located 4.3 kb downstream (chr16:75,577,708) of the reverse-oriented *CD46* gene in the ARS-UCD1.2 bovine reference assembly [chr16:75,582,021←75,621,057].

Off-target analyses revealed zero off-target sites matching the gRNA in the bovine reference genome (ARS-UCD1.2). Allowing for up to two mismatches and bulge sizes, 200 potential off-target sites were identified. Manual inspection of each of these sites with IGV showed no genomic sequence differences between the unedited and edited genomes except for the intended *CD46* edit. Thus, any phenotypic differences observed in BVDV susceptibility between unedited and edited calves can be confidently attributed to the on-target *CD46* edit rather than off-target modifications.

### 3.4. CD46 Expression and Localization

**Figure 3:**
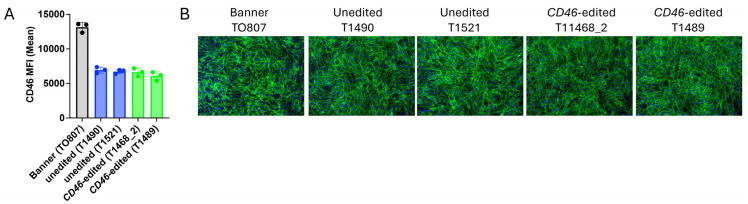
CD46 expression and localization in primary skin fibroblasts. Panel (**A**), Flow cytometric quantification of CD46 surface expression. Panel (**B**), Immunofluorescence staining of CD46 (green) and nuclei (blue; 20× magnification).

Primary skin fibroblasts derived from Banner’s clones showed approximately 50% of the CD46 surface protein expression observed in fibroblasts derived directly from Banner himself. Importantly, CD46 expression did not differ significantly between the cloned *CD46* edited and unedited control calves (Figure 3A). CD46 protein localization was also unaltered in *CD46* edited cells compared to unedited controls (Figure 3B). Thus, the *CD46* gene edit did not appreciably alter CD46 protein expression or localization in cells from the cloned Angus calves.

### 3.5. Ex Vivo BVDV Susceptibility Testing

**Figure 4:**
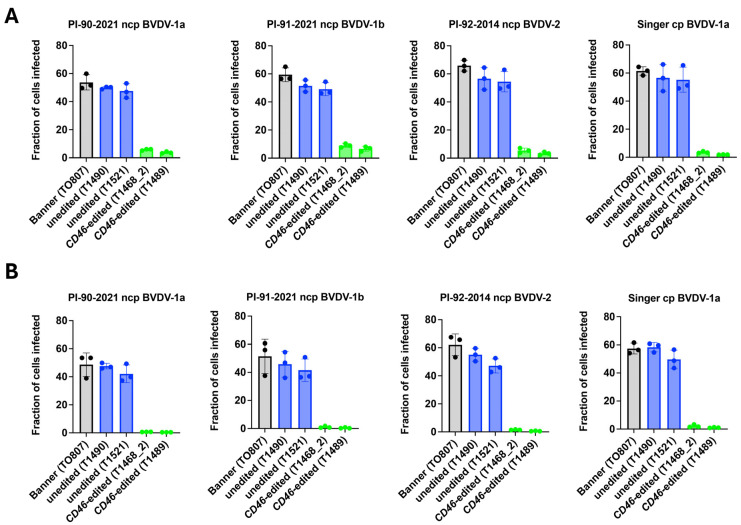
Comparison of BVDV Single-Step Growth in *CD46* Edited and Unedited Primary Skin Fibroblasts. Primary skin fibroblasts were inoculated with non-cytopathic (ncp) or cytopathic (cp) BVDV (panel (**A**)) or BVDV pre-incubated with heparin (panel (**B**)). BVDV infection was quantified by flow cytometry at 20 hpi.

Fibroblasts from *CD46* edited cloned calves showed dramatically reduced BVDV susceptibility, measured by flow cytometry at 20 h post-infection (hpi), compared to those directly from Banner or his unedited clones (Figure 4A). Given that BVDV utilizes heparan sulfate (HS) for CD46 independent entry, and that heparin can compete with HS for viral binding, the residual infectivity observed in *CD46* edited cells was investigated using heparin pre-treatment. Heparin pre-treatment abolished BVDV infection in *CD46* edited cells, indicating that the residual infectivity was likely mediated by HS (Figure 4B).

**Figure 5:**
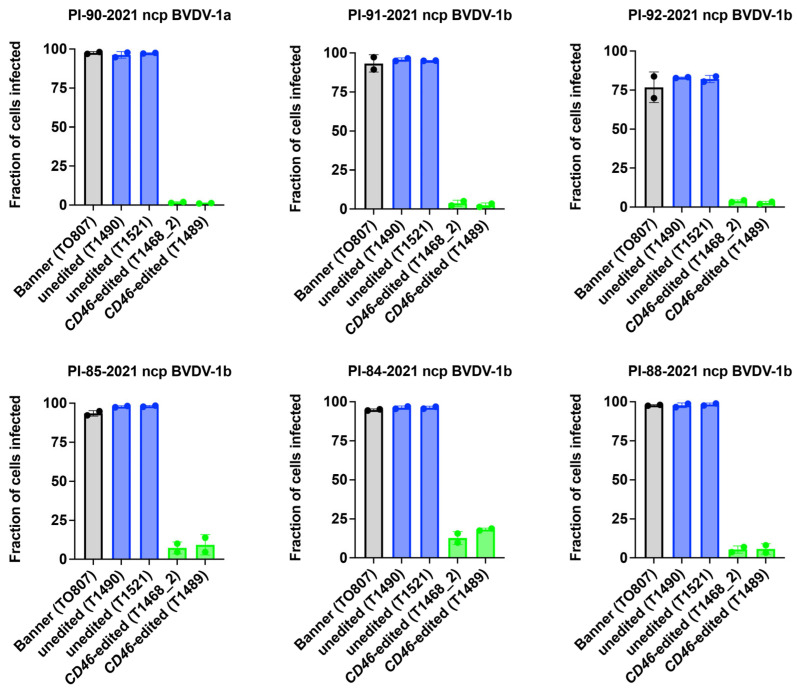
Comparison of BVDV Multi-Step Growth in *CD46* Edited and Unedited Primary Skin Fibroblasts. Primary skin fibroblasts were inoculated with serum collected from cattle persistently infected with non-cytopathic (ncp) BVDV. BVDV infection was quantified by flow cytometry at 72 hpi.

BVDV susceptibility was also assessed using non-cell culture adapted field strains. *CD46* edited cells showed a 92% average reduction (range: 81–99%) in susceptibility to BVDV field strains compared to unedited controls, as measured by flow cytometry at 72 h after inoculation with serum from persistently infected calves (Figure 5). Thus, the reduced BVDV susceptibility of the A_82_LPTFS edit in the male Angus (Banner) cloned primary skin fibroblasts replicated the finding in the female Gir (Ginger) cells, and suggests that editing for BVDV resistance is possible in cells from both Taurine and Indicine cattle.

## 4. Discussion

This report details the first attempt at producing American Angus calves carrying a targeted *CD46* gene edit. This genetic modification results in the expression of a novel receptor variant with a six amino acid substitution (G_82_QVLAL to A_82_LPTFS) in the BVDV binding domain. Although no cloned calves survived due to cloning-related morbidities, ex vivo BVDV susceptibility was assessed in primary skin fibroblasts. Consistent with previous findings in Gir cattle, the *CD46* edit conferred significant resistance to BVDV infection ex vivo, suggesting its potential for broad application in BVDV disease control.

Our in vitro, ex vivo, and in vivo studies have consistently demonstrated that a 19-nucleotide substitution within exon 2 of the *CD46* gene confers a significant reduction in susceptibility to BVDV infection [2]. The strong concordance observed between ex vivo infection studies and in vivo challenge outcomes suggests that the ex vivo results obtained with Angus fibroblast lines may also be predictive of in vivo responses to BVDV challenge. This correlation is particularly important since no live calves are available from the present study. It is worth noting that fibroblasts derived from both edited and unedited clones exhibited approximately 50% of the CD46 surface expression compared to the original Banner fibroblasts. While the key comparison between edited and unedited clones showed no difference, this reduction could potentially be a consequence of the cloning process or cell line derivation. Further investigation into the mechanisms underlying this observation may provide valuable insights into the cellular effects of cloning. Regardless, the successful application of this strategy in both *Bos indicus* (Gir) and *Bos taurus* (Angus) breeds underscores its potential for widespread use across various cattle breeds to reduce BVDV infections, mitigate economic losses, and improve animal welfare. While these results are promising, in vivo studies with live edited animals are needed to determine the magnitude and durability of resistance and the long-term efficacy of this approach in commercial cattle populations.

Despite rapid advancements in gene-editing technologies, efficiently producing live gene-edited animals remains a significant obstacle in cattle. Two popular approaches currently used are editing donor primary cells followed by SCNT or direct zygote editing. SCNT enables thorough characterization and selection of correctly modified cells prior to cloning, ensuring precise biallelic modifications. However, SCNT is limited by low efficiency, high costs, and health concerns, such as large offspring syndrome [18,19]. In contrast, direct zygote editing avoids cloning-related issues but introduces challenges like mosaicism, where different cells can carry distinct edits or even lack the intended modification entirely. Although techniques like trophoblast biopsies and preimplantation genetic testing can help detect mosaicism, current technological limitations hinder definitive confirmation of biallelic editing [20]. While selective breeding can eventually establish stable lines with germline transmission of desired edits, this process is resource-intensive and time-consuming. A third approach would be the use of bovine embryonic stem cell (bESC) lines made from stable primed pluripotent embryonic stem cells from bovine blastocysts [21]. However, reports of healthy live animals produced from this approach are not available in the scientific literature. Ultimately, improving the efficiency of producing gene-edited cattle will be necessary for broader adoption of this technology in the industry.

Together, the findings presented here underscore the potential of gene editing as a transformative tool for improving disease resistance in cattle, with significant implications for the livestock industry. The need for such interventions is clear, as BVDV imposes a major economic burden on the cattle sector, costing billions of dollars annually through reduced productivity, increased veterinary expenses, and immune suppression that leads to secondary infections [3,22]. The availability of cattle with genetically reduced susceptibility to BVDV could significantly lower disease prevalence, improve herd health, and reduce reliance on antibiotics. These outcomes contribute significantly to global agricultural sustainability efforts aimed at ensuring food security for a growing world population. Although scaling these technologies for wider testing and adoption remains challenging, continued advancements in gene editing and embryo production methods will help drive their successful application in commercial cattle populations.

## Figures and Tables

**Table 1:** Reproductive cloning outcomes and generation of primary skin fibroblast cell lines.

Status	Fetus/Calf ID	Abortion Date	Induction Date	Gestation Length (Days)	Weight (lbs)	Cell Line ID
Unedited	93345-A	-	7-Apr-2024	277	122	T1490
Unedited	93446F	-	-	283	112	T1521
*CD46* edited	87963F	5-Mar-2024	-	242	na	Failed
*CD46* edited	93361F	9-Mar-2024	-	246	77	T1468_2
*CD46* edited	88209-A	-	7-Apr-2024	275	98	T1489

na, not available, weight was not measured.

**Table 2:** WGS statistics and *CD46* exon 2 genotypes derived from Angus bull calf no. 202,118,042 (Banner).

								*CD46* Exon 2 Allele Counts ^a^			
Source	Sample Type	Edited Status	CD46 Genotype	Clone ID	Library ID	Sequence GB > Q20	Genome Coverage	Wt	Edited (a-Allele ^b^)	Edited (c-Allele)	Total
Bull calf	Blood	None	Wt	na	112586	70.0	23.3	9	0	0	9
Bull calf	Cell line	CD46	ALPTFS	3	116629	69.5	23.2	0	7	9	16
Bull calf	Cell line	CD46	ALPTFS	82	116423	39.1	13.0	0	5	3	8
Bull calf	Cell line	CD46	ALPTFS	156	116630	41.5	13.8	0	4	2	6
Cloned calf	Cell line	None	Wt	T1521	210221	60.1	20.0	18	0	0	18
Cloned calf	Cell line	None	Wt	T1490	210222	54.4	18.1	14	0	0	14
Cloned calf	Cell line	CD46	ALPTFS	T1489	210223	41.2	13.7	0	6	4	10
Cloned fetus	Cell line	CD46	ALPTFS	T1468	210224	40.8	13.6	0	1	3	4

^a^ Unmapped WGS reads identified by pattern match searching of unmapped FASTQ files with Linux environment command line searches and was independent of IGV scoring in BAM files. ^b^ The original *CD46* edit had an intentional synonymous a/c (“M”) SNP in the T85 codon. These are distinguished as a-allele and c-allele.

## Data Availability

Raw WGS files (fastq) for the primary cell lines are available in the NCBI SRA under accession number SRR33165226—SRR33165235. The sequence data have also been deposited with links to BioProject accession number PRJNA1245396 (BioSamples SAMN47753601, SAMN47754587, SAMN47755051, and SAMN48016586-SAMN48016592) in the NCBI BioProject database. In addition, IGV access to the aligned sequences (bam files and IGV session files) is available. IGV session link (IGV: [File] > [Load from URL]): https://s3.us-west-2.amazonaws.com/usmarc.heaton.public/WGS/AngusCD46/ARS1.2/sessions/AngusBanner_CD46_10tracks.xml (accessed on 1 March 2025).

## Supplementary Materials

The following supporting information can be downloaded at: https://www.mdpi.com/article/10.3390/v17060775/s1, File S1: Summary of Necropsy Reports.

## Abbreviations

The following abbreviations are used in this manuscript:

bESC	Bovine Embryonic Stem Cell
BVDV	Bovine Viral Diarrhea Virus
CRISPR	Clustered Regularly Interspaced Short Palindromic Repeats
HDR	Homology-Directed Repair
HS	Heparan Sulfate
IGV	Integrative Genomics Viewer
RFLP	Restriction Fragment Length Polymorphism
SCNT	Somatic Cell Nuclear Transfer
SNP	Single Nucleotide Polymorphism
ssODN	Single-Stranded Oligodeoxynucleotide
WGS	Whole Genome Sequencing
