# Supplementary material for: CD46 Gene Editing Confers Ex Vivo BVDV Resistance in Fibroblasts from Cloned Angus Calves
Source: Viruses. 2025 May 29;17(6):775. doi: 10.3390/v17060775 (PMC12197525; doi:10.3390/v17060775)
Supplement: Supplementary file 1 [file viruses-17-00775-s001.zip › viruses-3649422-supplementary.pdf]

Supplementary File S1. Summary of Necropsy Reports

Animal ID: 93361F, *CD46*-edited & cloned
This *CD46*-edited and cloned calf, aborted late in gestation, presented with significant and likely fatal liver abnormalities (hepatopathy) strongly suggestive of a primary vascular developmental issue, accompanied by a substantial abdominal effusion. While fetal aspiration and thymic hemorrhage were also present, the liver pathology appears to be the primary and most striking finding. Infectious causes were ruled out by PCR and bacterial culture (BVDV, IBR, *Leptospira*, *Neospora*).

Animal ID: 88209-A, *CD46*-edited & cloned
This *CD46*-edited and cloned stillborn calf presented with a complex and widespread array of congenital anomalies, most notably severe and generalized musculoskeletal abnormalities (arthrogryposis, myopathy with apparent hypertrophy) affecting skeletal, diaphragmatic, and cardiac muscle. Significant cardiac, cranial, renal, and liver pathologies were also identified. Infectious causes were ruled out (BVDV). This case highlights a severe, multi-systemic developmental failure.

Animal ID: 93345-A, Unedited & cloned
This unedited, cloned calf, which died shortly after birth, presented with significant congenital skeletal deformities, a notable fluid-filled liver cyst, and generalized gross muscle pallor. There was evidence of fetal aspiration and likely ingestion of a blue-tinted substance, possibly an oral supplement or medication administered via tube feeding. While some findings (e.g., liver glycogen) were noted as potentially normal for a neonate, the combination of structural abnormalities and the liver cyst are significant. Infectious causes were ruled out (BVDV).

Animal ID: 93446F, Unedited & cloned
This unedited, cloned calf, which died shortly after birth, exhibited a severe, multi-systemic hemorrhagic diathesis, with bleeding observed extensively in muscles, subcutaneous tissues, meninges, lungs, and various organs. The presence of abundant blue fluid throughout the respiratory and digestive tracts suggests significant aspiration and/or ingestion of a blue-tinted substance, potentially from tube feeding or medication attempts. The enlarged, pale, and soft liver is also a notable finding, consistent with reports in cloned animals. While infectious causes were ruled out (BVDV), the pathologist suggested parturition trauma or tube feeding as potential contributors to some hemorrhagic and aspiration lesions.
